# Development of Resistance to Pyrethroid in *Culex pipiens pallens* Population under Different Insecticide Selection Pressures

**DOI:** 10.1371/journal.pntd.0003928

**Published:** 2015-08-14

**Authors:** Linna Shi, Hongxia Hu, Kai Ma, Dan Zhou, Jing Yu, Daibin Zhong, Fujin Fang, Xuelian Chang, Shengli Hu, Feifei Zou, Weijie Wang, Yan Sun, Bo Shen, Donghui Zhang, Lei Ma, Guofa Zhou, Guiyun Yan, Changliang Zhu

**Affiliations:** 1 Department of Pathogen Biology, Nanjing Medical University, Nanjing, China; 2 Department of Pathogenic Microorganism and Laboratory Medicine, Shaanxi University of Chinese Medicine, Shaanxi, China; 3 Program in Public Health, College of Health Sciences, University of California at Irvine, Irvine, California, United States of America; Johns Hopkins Bloomberg School of Public Health, UNITED STATES

## Abstract

Current vector control programs are largely dependent on pyrethroids, which are the most commonly used and only insecticides recommended by the World Health Organization for insecticide-treated nets (ITNs). However, the rapid spread of pyrethroid resistance worldwide compromises the effectiveness of control programs and threatens public health. Since few new insecticide classes for vector control are anticipated, limiting the development of resistance is crucial for prolonging efficacy of pyrethroids. In this study, we exposed a field-collected population of *Culex pipiens pallens* to different insecticide selection intensities to dynamically monitor the development of resistance. Moreover, we detected *kdr* mutations and three detoxification enzyme activities in order to explore the evolutionary mechanism of pyrethroid resistance. Our results revealed that the level of pyrethroid resistance was proportional to the insecticide selection pressure. The *kdr* and metabolic resistance both contributed to pyrethroid resistance in the *Cx*. *pipiens pallens* populations, but they had different roles under different selection pressures. We have provided important evidence for better understanding of the development and mechanisms of pyrethroid resistance which may guide future insecticide use and vector management in order to avoid or delay resistance.

## Author Summary

Successful population control of mosquitoes is key to preventing transmission and epidemics of mosquito-borne diseases. This strategy relies heavily on insecticides, particularly pyrethroids. However, widespread pyrethroid resistance has hindered vector control implementation and sustainability. Generally, pyrethroid resistance in insects is mainly caused by target-site insensitivity and metabolic resistance. Although studies on the two resistance mechanisms have provided insights, how the target-site and metabolic resistance mechanisms jointly confer the resistance phenotype has remained unclear. Understanding the mechanism of resistance to insecticides is essential for vector control measures. Our study investigated the development of resistance to a pyrethroid in three mosquito lines by varying the intensity of selection between lines (intense selection, mild selection and no selection) and then tracking changes in *kdr* frequency and the activities of three families of metabolic detoxification enzymes over time. These findings may lead to the development of more appropriate use of insecticides and more accurate resistance monitoring systems in the field.

## Introduction

*Culex* mosquitoes are important vectors responsible for transmission of lymphatic filariasis (LF) and several viral pathogens to millions of people worldwide, including St. Louis encephalitis, West Nile encephalitis, eastern equine encephalitis, Venezuelan equine encephalitis and Japanese encephalitis [1]. The World Health Organization estimated over 120 million cases of LF and about 40 million disfigured and incapacitated by the disease [2]. Globally, nearly 1.4 billion people in 73 countries worldwide are currently threatened by LF. Mosquito-borne diseases dramatically affect public health and pose a major burden in terms of economy and development worldwide. Mass drug administration in combination with alternative vector control methods have proven to be more effective and practical in avoiding the re-emergence and re-introduction of LF [3, 4]. Insect control is the primary intervention available for some of the most devastating mosquito-borne diseases, particularly those lacking vaccines such as malaria, dengue and LF [5]. Most vector control programs largely rely on the application of chemical insecticides by the use of outdoor spraying, insecticide-treated nets (ITNs) or indoor residual spraying (IRS) [6]. Because of the relatively low mammalian toxicity and rapid knockdown effect on insects, pyrethroids are the most commonly used insecticides and constitute the only recommended class of insecticides for ITNs. However, insecticide exposure is a potent selective force, presenting a risk of generating resistance that would threaten the efficacy of control programs. Hence, preventing or delaying the emergence and development of resistance to pyrethroids is very important for vector control efforts. Improving vector management involves a better understanding of resistance mechanisms.

Global surveys have indicated that resistance of mosquitoes to pyrethroids mainly occurs through increased detoxification, as well as target site insensitivity [7]. ‘Metabolic resistance’ usually results from enhanced detoxification enzyme activity in resistant organisms [8]. Detoxification enzymes typically linked to insecticide resistance mainly include three major gene families, cytochrome P450 monooxygenases (P450s or CYPs), carboxyl/choline esterases (CCEs) and glutathione-S-transferases (GSTs). Numerous studies have associated these detoxification enzymes with pyrethroid resistance in mosquitoes [9–11]. The primary target sites of pyrethroids, well known as knockdown resistance (*kdr*), encode voltage-gated sodium channels, and single or multiple substitutions in the sodium channel gene can reduce neuronal sensitivity to pyrethroids [12]. To date, More than 30 unique resistance-associated *kdr* mutations or combinations of mutations have been detected in several insect species [13]. Among them, the most common *kdr* mutations are the leucine to phenylalanine (Leu→Phe) substitution and the leucine to serine substitution (Leu→Ser) substitution at codon 1014 in the S6 hydrophobic segment of domain II in the sodium channel gene [14, 15]. These two common mutations have been shown to reduce the pyrethroid sensitivity of sodium channels in *Xenopus* oocytes, confirming their role in *kdr* [16, 17]. Many studies have revealed that all these mechanisms can occur simultaneously in resistant populations with cumulative phenotypic effects leading to resistance to a single or multiple insecticides [18, 19]. However, the relative contribution of the metabolic resistance and knockdown resistance in conferring the resistance phenotype has remained elusive.

High levels of resistance to pyrethroids in *Culex* mosquitoes has been widely reported [20, 21]. Here, we studied *Cullex pipiens pallens* because it is the most prevalent and important vector in China with high population density. We also chose deltamethrin, a representative pyrethroid insecticide, to explore the evolutionary mechanism of insecticide resistance, as well as the relative contributions of the target-site and metabolic resistance in the development of pyrethroid resistance. We detected *kdr* and activities of three types of detoxification enzymes (P450s, CCEs, and GSTs) in the same mosquito sample through large-scale population studies. Knowledge of the changing trends and patterns of insecticide resistance may impact future predictions and monitoring of pyrethroid resistance in mosquitoes and other arthropod pests and disease vectors.

## Materials and Methods

### Set-up of mosquito population

A population of *Cx*. *pipiens pallens* was collected from natural habitats (Tangkou, Shandong Province of China) in September 2009. Mosquitoes were reared in standard insectary conditions (28°C, 14 h:10 h light/dark period, 75% relative humidity) with tap water (larvae) and net cages (adults). Adults were supplied with a 10% sucrose solution and blood fed on adult mice. Larvae were fed with fish food.

The population was selected with the pyrethroid insecticide deltamethrin (Jiangsu Yangnong Chemical Group Co., Jiangsu, China) in the laboratory. Selection was performed by exposing each generation of fourth-stage larvae for 24 h to a 50% lethal concentration (LC_50_) of deltamethrin. The LC_50_ was determined by a larval bioassay. Initially, the fourth-stage larvae were exposed to a wide range of test concentrations of deltamethrin. After determining the mortality of larvae in this wide range of concentrations, a narrower range (of 5 concentrations, yielding between 5% and 95% mortality in 24 h) was used to determine LC_50_ values. Five concentrations of deltamethrin and 3 replicates of 20 fourth-instar larvae per concentration were used. A control group was measured using 20 larvae in tap water without any insecticides. Numbers of dead and surviving larvae were recorded after 24 h. All surviving larvae were transferred to tap water, fed with larval food and allowed to emerge. Adults were fed to obtain eggs for the next generation.

After six generations, three strains under different insecticide selection pressures were established. The first strain, designated the ‘intense selection’ (IS) strain, was selected with the LC_50_ of deltamethrin for each generation causing 50–60% larval mortality. The second selected strain, termed the ‘mild selection’ (MS) strain, was exposed to the constant concentration of 0.05 ppm (LC_50_ for generation 6) in the subsequent deltamethrin selection. Deltamethrin exposure was withdrawn from the last strain, which was designated the ‘no-selection’ (NS) strain. All three strains were selected for 24 generations with three replicate groups, and each generation of every replicate group was initiated with more than 1000 adult females in order to limit bottleneck effects. The actual doses used for deltamethrin selection per generation were reported in the supporting information (detailed results are shown in S1 Table). A laboratory deltamethrin-susceptible strain of *Cx*. *pipiens pallens* (S-LAB) was used as a reference strain.

### Sample collection

In every replicate group of each strain, more than 50 female adult mosquitoes, at post-emergence ages of 3–4 d, were collected for determinations of *kdr* alleles and metabolic enzyme activities. Generations 1, 6, 10, 14, 18, 22, 26 and 30 were analyzed. Two legs of each adult female were removed and preserved individually in 95% alcohol for subsequent DNA analysis. The remaining mosquito body was immediately used for metabolic enzyme activity determination. More than 3,000 female adult mosquitoes were used for analysis in this study.

### DNA extraction and *kdr* allele detection

Genomic DNA was extracted individually from two legs of each adult female by a fast tissue-to-PCR kit (Fermentas, K1091). The region containing *kdr* mutations within the *para* sodium channel gene was amplified by PCR with primers Cpp1 (5’-CCTGCCACGGTGGAACTTC-3’) and Cpp-2 (5’-GGACAAAAGCAAGGCTAAGAA-3’). The PCR primers were designed based on the cDNA sequence of *Cx*. *quinquefasciatus para*-sodium channel gene alpha subunit (Genbank accession number BN001092) [22]. PCR amplification was carried out in a volume of 20 μl, including 4 μl genomic DNA, 10 μl Tissue Green PCR Master Mix (Fermentas), 10 pmol primers Cpp1 and Cpp2 and 4 μl nuclease-free water. Amplification was performed with the following cycling conditions: initial denaturation at 95°C for 3 min, 35 cycles of 95°C for 30 s, 55°C for 30 s and elongation at 72°C for 40 s, followed by extension at 72°C for 3 min. PCR products were purified using the QIAquick PCR purification kit (Qiagen) and then sent for sequencing (BGI, Shanghai, China). In total, 2,427 female adult samples were successfully sequenced in the study. Genotype frequencies were calculated, and deviation from the Hardy–Weinberg equilibrium was analyzed by the web-based program GENEPOP [23].

### Metabolic enzyme activity assays

Metabolic enzyme activity was measured in individual female mosquitoes by using the method described by Daibin *et al*. [24] with a slight modification. Every individual females was homogenized in a 1.5-ml tube with 300 μl of 0.25 M phosphate buffer (pH 7.2) and diluted by adding 300 μl of phosphate buffer. The tube was mixed and centrifuged, and the same supernatant was used to test the activity of GSTs, P450s and CCEs simultaneously. All assays were carried out in duplicate, and the protein content of the supernatant was measured by the Bradford method [25]. For the GSTs activity assay, a total of 90 μl of reduced glutathione solution (Sigma, G4251) and 90 μl of 1-chloro-2,4'-dinitrobenzene (cDNB) solution was added to 90 μl of mosquito supernatant. The absorbance was measured immediately using a microplate reader at 340 nm and then detected every 2 min for five times, using 0.25 M phosphate buffer as the negative control. For the P450s activity assay, a total of 10 μl of the 60mM 7-ethoxycoumarine (7-EC) solution was added to 100 μl of mosquito supernatant, and samples were incubated at 30°C for 5.5 h. The reaction was stopped by the addition of 150 μl of glycine buffer (pH 10.4, 1 mM), and sample absorbance was measured using a microplate reader at 450 nm with 0.25 M phosphate buffer as a negative control. The OD values were converted into concentrations by using standard regression based on a serial dilution of 7-hydroxycoumarin and its relevant OD values. The content of P450s was calculated for each mosquito. The OD values were converted into concentrations by using standard regression based on OD values of serial dilutions of 7-hydroxycoumarine. For the CCEs activity assay, the 0.1 mM β-nitrophenyl acetate (Sigma, N8130) solution was placed for 5 min in 30°C water bath first, and then a total of 220 μl of the β-nitrophenyl acetate solution was added to 50 μl of mosquito supernatant. The absorbance was measured using a microplate reader at 405 nm every min for five times with 0.25 M phosphate buffer as the negative control. All measurements were performed in duplicate. In total, we successfully detected 1,541 female adult samples for P450s, 1,368 samples for CCEs and 1,665 samples for GSTs.

### Statistical analysis

The LC_50_ was calculated using Probit analysis [26] and Abbott’s correction for the mortality rate in the control group [27]. The resistance ratio (RR) is the ratio of the LC_50_ of the selected strain to the LC_50_ of the laboratory deltamethrin-susceptible strain S-LAB. Regression analysis (Curve estimation) was used to determine correlation coefficients between the deltamethrin susceptibility and generations under different selection. The DNA sequences of *kdr* were assembled and prealigned by BioEdit, aligned in ClustalW implemented in MEGA5 and the alignment was refined manually [28]. Then we used DnaSPv5 to estimate the number of haplotypes (h), the haplotype diversity (Hd) and nucleotide diversity (Pi). The *kdr* allele frequency in three selected strains was calculated, and statistically significant differences among strains were examined using the *t*-test. One-way analysis of variance (ANOVA) was used to examine whether metabolic enzyme activity varied in populations with different selection pressures. The generation was treated as a random factor. Metabolic enzyme activity data were square root transformed. The *t*-test was used for comparison of different generations when appropriate. Linear correlation analysis was used to study the correlation of the frequency of *kdr* and the metabolic enzyme activity with the degree of resistance.

### Accession numbers

*Cx*. *quinquefasciatus para*-sodium channel gene alpha subunit: BN001092.

*Cx*. *pipiens pallens kdr* haplotypes: GU198936- GU198938, GU339221.

## Results

### Mosquito populations and deltamethrin susceptibility

**Fig 1:**
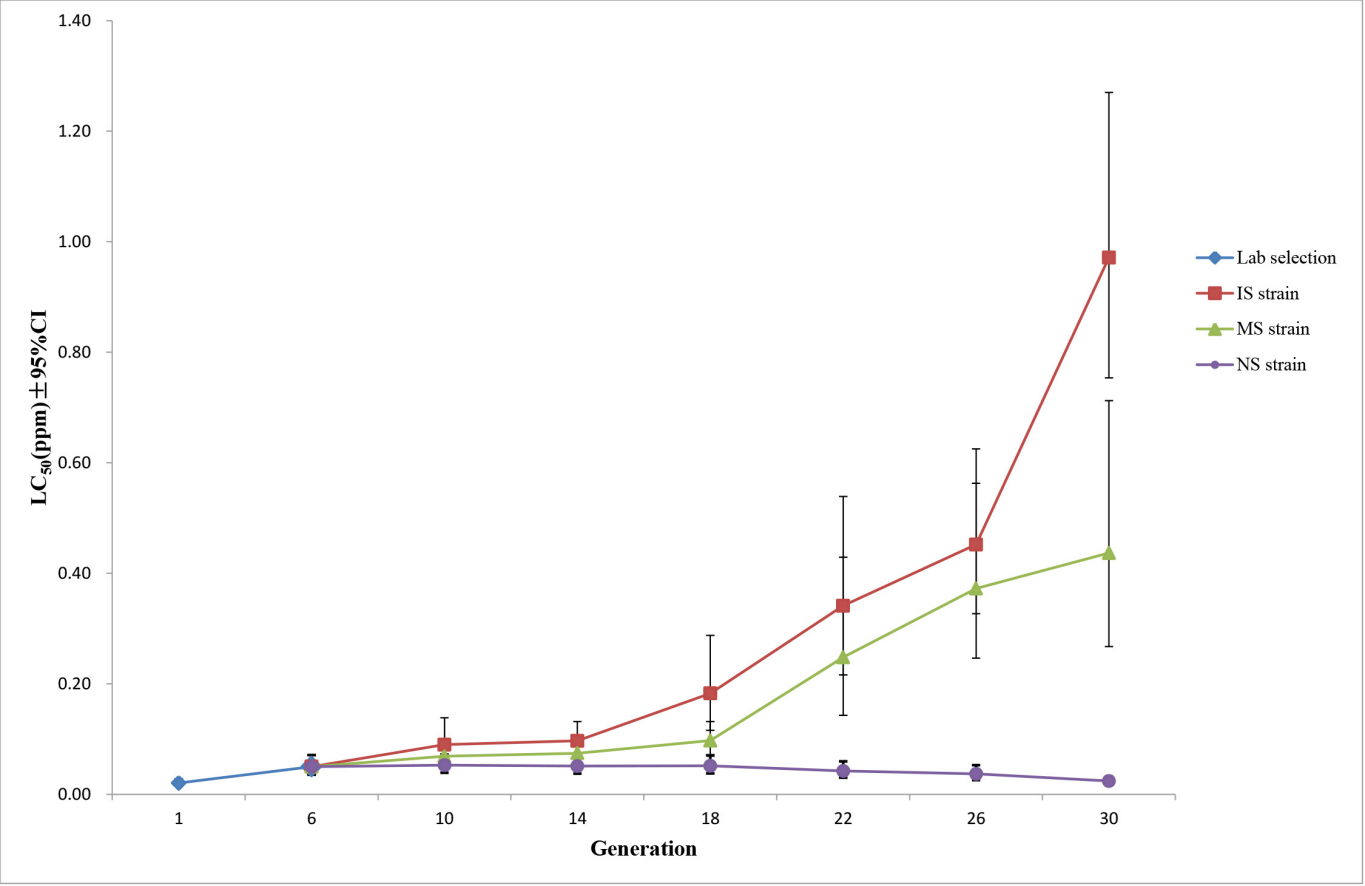
Development of larval resistance of populations under different deltamethrin selection pressures. Levels of deltamethrin resistance were dynamically monitored in three selected strains. Lab selection: the population from 1 to 6 generations were selected with insecticide together and the selection concentration was LC_50_ of deltamethrin for each generation. IS strain: selection concentration was LC_50_ of deltamethrin for each generation and resulted in 50–60% larval mortality. MS strain: selection concentration was the LC_50_ of deltamethrin for generation 6 (0.05 ppm), and the larval mortality was gradually decreased. NS strain: exposure to deltamethrin was withdrawn after generation 6. CI: confidence intervals.

**Fig 2:**
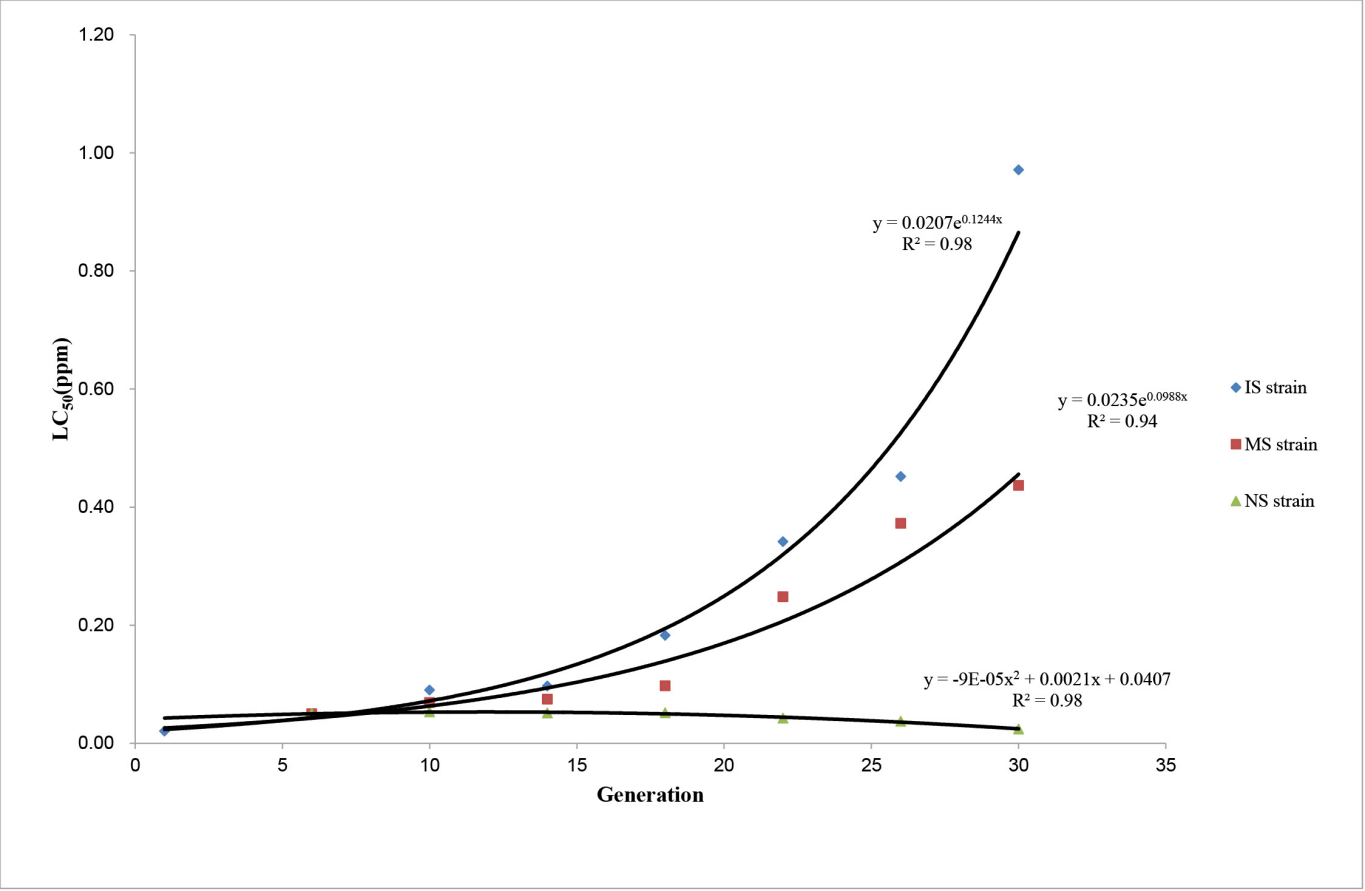
Scatter plot of the level of resistance and generation under different insecticide selection. In the IS strain, R^2^ = 0.98, *P*<0.0001; In the MS strain, R^2^ = 0.94, *P*<0.0001; In the NS strain, R^2^ = 0.98, *P*<0.0001.

A field population of *Cx*. *pipiens pallens* was collected, and three strains were established after being placed for 30 generations (~28 months) under different insecticide selection pressures. After selection for six generations, the RR increased from 1.69 at generation 1 (LC_50_ = 0.0206 ppm) to 4.11 at generation 6 (LC_50_ = 0.0501 ppm) (detailed results are shown in S2 Table). The resistance to insecticide was increased from generation to generation with exposure to insecticide, while it was reduced without exposure (Fig 1). Regression analysis showed that the level of resistance grew exponentially in IS and NS strain (Fig 2). The degree of insecticide resistance in the IS strain increased more rapidly than that in the MS strain. At generation 30, the RR of the IS strain had increased significantly to 79.61 (LC_50_ = 0.9713 ppm) (*P* < 0.05), and the RR of the MS strain was also increased significantly to 35.80 (LC_50_ = 0.4367 ppm) (*P* < 0.05). Notably, the level of insecticide resistance increased faster after generation 14 of the IS strain and generation 18 of the MS strain. By contrast, the level of insecticide resistance in the NS strain was reduced slowly without insecticide selection from generation 6, and significant differences were observed after generation 22. The RR of the NS strain was reduced significantly from 4.11 at generation 6 (LC_50_ = 0.0501 ppm) to 1.98 at generation 30 (LC_50_ = 0.0241 ppm) (*P* < 0.05), and the LC_50_ of generation 30 was still higher than that of the first generation (*P* < 0.05) (Fig 1).

### Distribution of *kdr* allele frequencies in populations with different selection pressures

**Fig 3:**
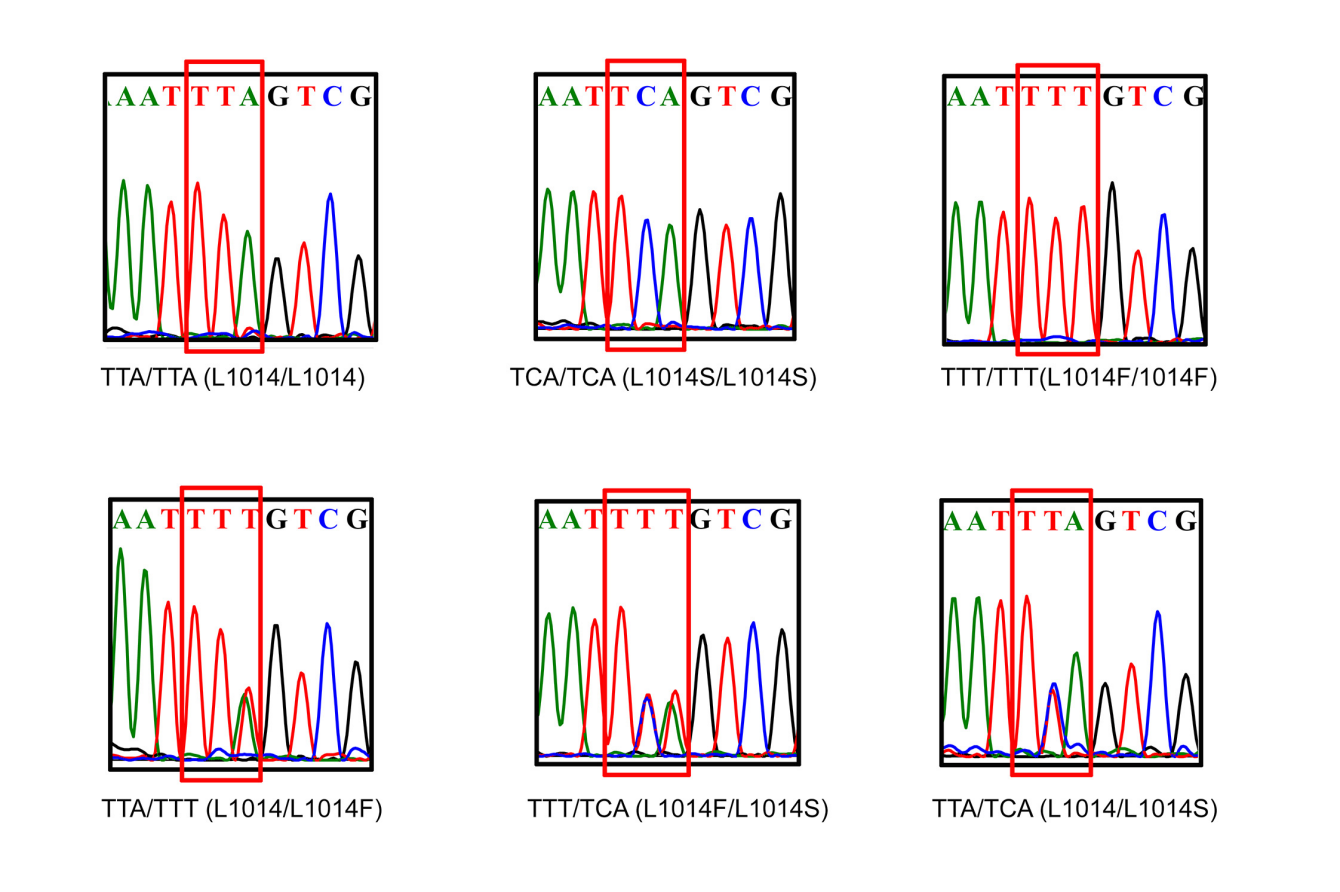
Population *kdr* genotypes. A total of six genotypes were identified in this *Cx*. *pipiens pallens* population: TTA/TTA, TCA/TCA, TTT/TTT, TTA/TTT, TTT/TCA and TTA/TCA. Red box indicates the 1014 site of the *kdr* gene.

**Fig 4:**
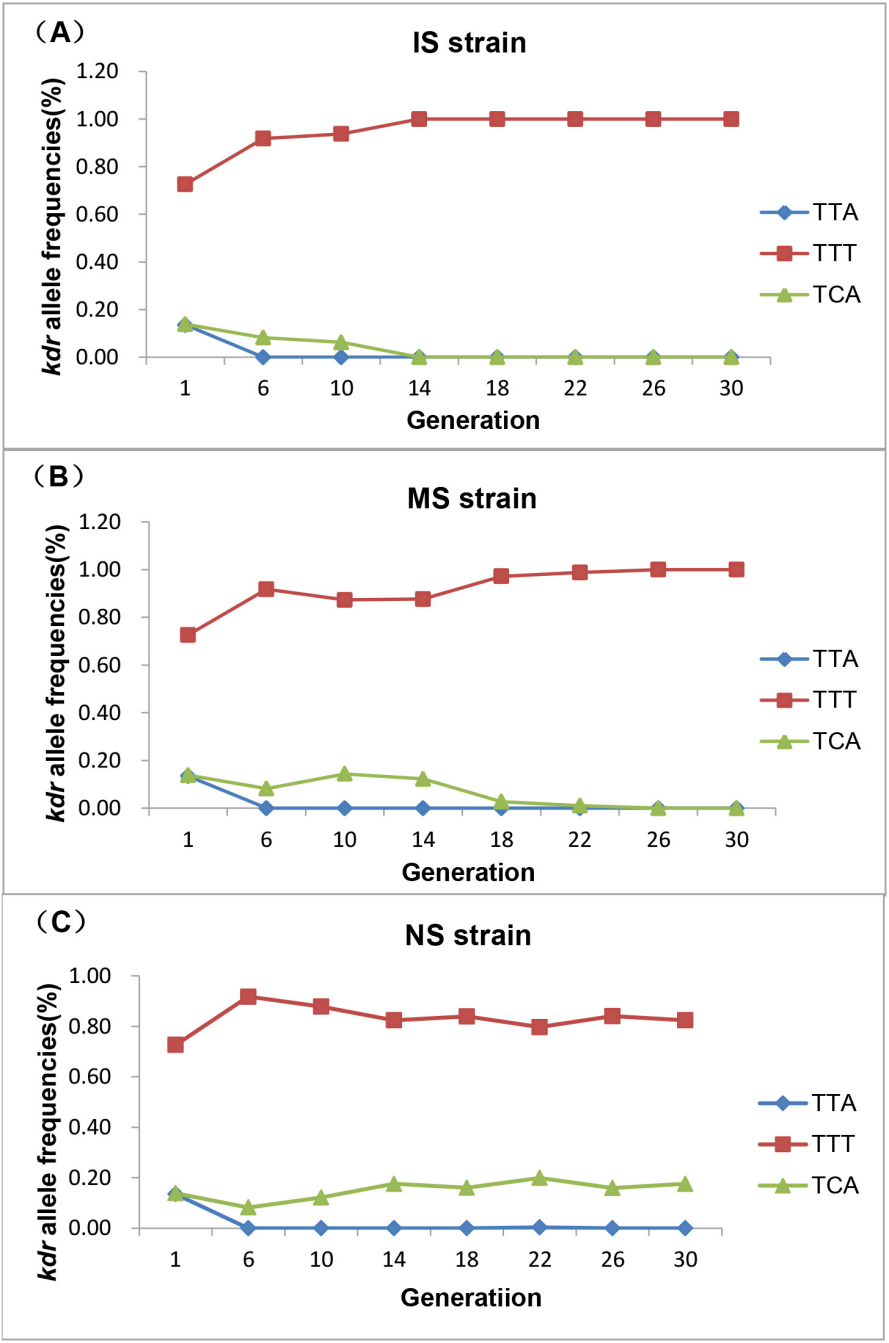
Development of *kdr* allele frequencies in populations with different deltamethrin selection pressures. Dynamic variation of three *kdr* allele frequencies are shown for (A) IS strain, (B) MS strain, and (C) NS strain.

A 480-bp fragment of the para-type sodium channel gene including codon 1014 was sequenced from 2,457 individual *Cx*. *pipiens pallens* of the three strains. The wild-type *kdr* codon sequence spanning position 1014 was TTA (L1014). The two most common types of *kdr* mutations detected were TTT (L1014F) and TCA (L1014S). A total of six genotypes were identified in all strains: L1014/L1014, L1014F/L1014F, L1014S/L1014S, L1014/L1014F, L1014/L1014S and L1014F/L1014S (Fig 3). The field population of *Cx*. *pipiens pallens* carried a high *kdr* mutation frequency (86.49%) of both the L1014F (72.64%) and L1014S (13.85%) mutations (Table 1). Under insecticide selection, the *kdr* mutation frequency increased significantly and reached 100.00% at generation 6. But the response of these two mutations to deltamethrin selection were different (Table 1 and Fig 4). Under insecticide selection, the frequency of L1014F was increased and faster in IS strain than MS strain. By contrast, the frequency of L1014S was decreased and slower in MS strain than IS strain. In the IS strain, the frequency of the L1014F mutation became fixed, and the strain became homozygous for *kdr* (genotype: L1014F/L1014F) at generation 14. In the MS strain, the rate of increase in the allele frequency for this mutation was slower than observed for the IS strain, and the MS strain became homozygous (genotype: L1014F/L1014F) at generation 26. Interestingly, the level of insecticide resistance was increased most significantly after the population became a homozygous population for the *kdr* resistance gene, suggesting the existence of other mechanism which could induce deltamethrin resistance. Without insecticide selection, the frequency of L1014S increased and L1014F decreased. In the NS strain, the frequency of L1014S increased significantly from 8.21% at generation 6 to 17.73% at generation 30, and the frequency of L1014F declined significantly but was still higher than that of the first generation (72.64%). These results suggest that the L1014F mutation is more closely associated with deltamethrin resistance in the *Cx*. *pipiens pallens* population than the L1014S mutation. The wild-type L1014 allele could not be detected from the sixth generation and became extinct with insecticide selection. Because our studied population carried a high *kdr* mutation frequency and was a closed population, the wild-type sequence was recovered infrequently. The three replicate groups had the same trends, and the detailed results are shown in S1 Fig.

**Table 1:** Genotypes and allele frequencies of *kdr* detected in populations under different insecticide selection pressures.

			*kdr genotypes*						*Kdr alleles frequencies(%)*			*kdr mutation frequency(%)*
Strains	Generation	N	TTA/TTA	TTT/TTT	TCA/TCA	TTA/TTT	TTA/TCA	TTT/TCA	TTA	TTT	TCA	TTT+TCA
**Lab**	**1**	148	9	84	6	20	2	27	13.51	72.64	13.85	86.49
**selection**	**6**	134	0	120	8	0	0	6	0.00	91.79	8.21	100.00
**IS**	10	133	0	118	3	0	0	12	0.00	93.23	6.77	100.00
**strain**	14	102	0	102	0	0	0	0	0.00	100.00	0.00	100.00
	18	143	0	143	0	0	0	0	0.00	100.00	0.00	100.00
	22	97	0	97	0	0	0	0	0.00	100.00	0.00	100.00
	26	94	0	94	0	0	0	0	0.00	100.00	0.00	100.00
	30	109	0	109	0	0	0	0	0.00	100.00	0.00	100.00
**MS**	10	109	0	88	8	0	0	15	0.00	87.61	14.22	100.00
**strain**	14	112	0	89	5	0	0	18	0.00	87.50	12.50	100.00
	18	139	0	133	2	0	0	4	0.00	97.12	2.88	100.00
	22	104	0	103	1	0	0	0	0.00	99.04	0.96	100.00
	26	127	0	127	0	0	0	0	0.00	100.00	0.00	100.00
	30	104	0	104	0	0	0	0	0.00	100.00	0.00	100.00
**NS**	10	114	0	95	9	0	0	10	0.00	87.72	12.28	100.00
**strain**	14	107	0	78	8	0	0	21	0.00	82.71	17.29	100.00
	18	133	0	98	8	0	0	27	0.00	83.83	16.17	100.00
	22	143	0	100	14	1	0	28	0.35	80.07	19.58	99.65
	26	140	0	106	11	0	0	23	0.00	83.93	16.07	100.00
	30	141	0	100	9	0	0	32	0.00	82.27	17.73	100.00

### Patterns of haplotype variation

The haplotype number (K) and haplotype diversity (H) are informative statistics for describing the distribution of haplotypes under an infinite-sites model [29]. During a selective sweep, the reduction in variation around a naturally selected locus will reduce the impact of reshuffling by recombination producing new haplotypes, and recombination is more likely to occur between two copies of the same haplotype [30]. In this study, the intron region downstream of L1014 in the *kdr* gene was cloned, sequenced then analyzed using DNAspv5 software. The results showed a significant reduction of K and H in the *kdr* gene under insecticide selection, and the nucleotide diversity (Pi) and haplotype diversity (Hd) decreased with the increase of resistance (Table 2). Pi and Hd were reduced to 0 at generation 26 in the IS strain and at generation 30 in the MS strain, but they were increased after withdrawal of deltamethrin.

**Table 2:** DNA polymorphisms of *kdr* gene in populations under different insecticide selection pressures.

Strain	Generation	Number of sequences	Number of sites	h	Hd	Pi
Lab Selection	1	66	480	39	0.901	0.09702
	6	81	480	17	0.513	0.04526
IS strain	10	62	480	13	0.45	0.02003
	14	65	480	11	0.374	0.04052
	18	66	480	9	0.281	0.00778
	22	57	480	2	0.064	0.00014
	26	53	480	1	0	0
	30	62	480	1	0	0
MS strain	10	72	480	15	0.438	0.01436
	14	86	480	14	0.281	0.00422
	18	76	480	13	0.356	0.01741
	22	63	480	5	0.124	0.00104
	26	57	480	3	0.07	0.01673
	30	64	480	1	0	0
NS strain	10	65	480	20	0.638	0.03574
	14	76	480	24	0.752	0.04275
	18	71	480	28	0.725	0.04657
	22	66	480	34	0.832	0.09714
	26	78	480	32	0.876	0.08175
	30	68	480	36	0.891	0.0398

h: Number of Haplotypes, Hd: Haplotype diversity, Pi: Nucleotide diversity.

### Metabolic enzyme assay

The field population of *Cx*. *pipiens pallens* was selected with deltamethrin in the laboratory due to unknown use of insecticides at the sampling site. Metabolic enzyme activities were detected after deltamethrin selection for six generations. We analyzed in total 1545 samples for P450s, 1371 samples for CCEs and 1653 samples for GSTs (Table 3).

**Table 3:** Dynamic activities of three detoxification enzymes in populations under different insecticide selection pressures.

		P450s activity			CCEs activity			GSTs activity		
Strain	Generation	mean(ng/min/mg protein)	SD	N	mean(nmol/min/mg protein)	SD	N	mean(nmol/min/mg protein)	SD	N
	6	1777.19	947.73	54	42.78	23.12	47	10.86	3.52	49
IS	10	2086.80	938.54	87	44.19	28.01	66	9.13	4.17	98
strain	14	2075.14	871.51	86	46.10	28.55	72	9.88	5.11	69
	18	2354.70	1058.67	96	49.78	28.64	70	11.31	5.43	98
	22	2727.20	1118.18	88	59.73	27.06	80	9.35	4.50	97
	26	2768.20	1330.72	84	62.97	37.32	80	9.87	3.93	97
	30	3941.26	1223.07	72	66.17	37.29	83	12.07	7.13	96
MS	10	2184.01	693.65	89	41.07	23.08	72	13.64	7.25	87
strain	14	2092.73	986.13	96	41.14	25.54	82	16.28	9.72	88
	18	2162.80	1057.03	86	43.12	26.16	70	9.56	5.53	88
	22	2241.57	926.78	88	41.74	29.85	71	11.48	3.90	88
	26	2287.26	1042.15	78	45.31	29.00	85	11.19	3.77	87
	30	2873.73	1080.77	77	49.37	34.12	83	12.92	5.02	89
NS	10	1939.10	665.47	90	37.38	28.42	72	8.42	3.22	78
strain	14	1950.98	565.64	84	36.81	24.29	63	10.92	4.85	90
	18	1692.62	564.18	69	34.81	22.30	77	8.09	3.17	89
	22	1795.91	563.41	74	31.02	20.69	70	9.02	2.99	88
	26	1570.71	701.16	83	28.65	15.67	61	9.39	3.37	88
	30	1438.93	531.46	64	25.16	12.51	67	10.73	3.37	89
SUM				1545			1371			1653

**Fig 5:**
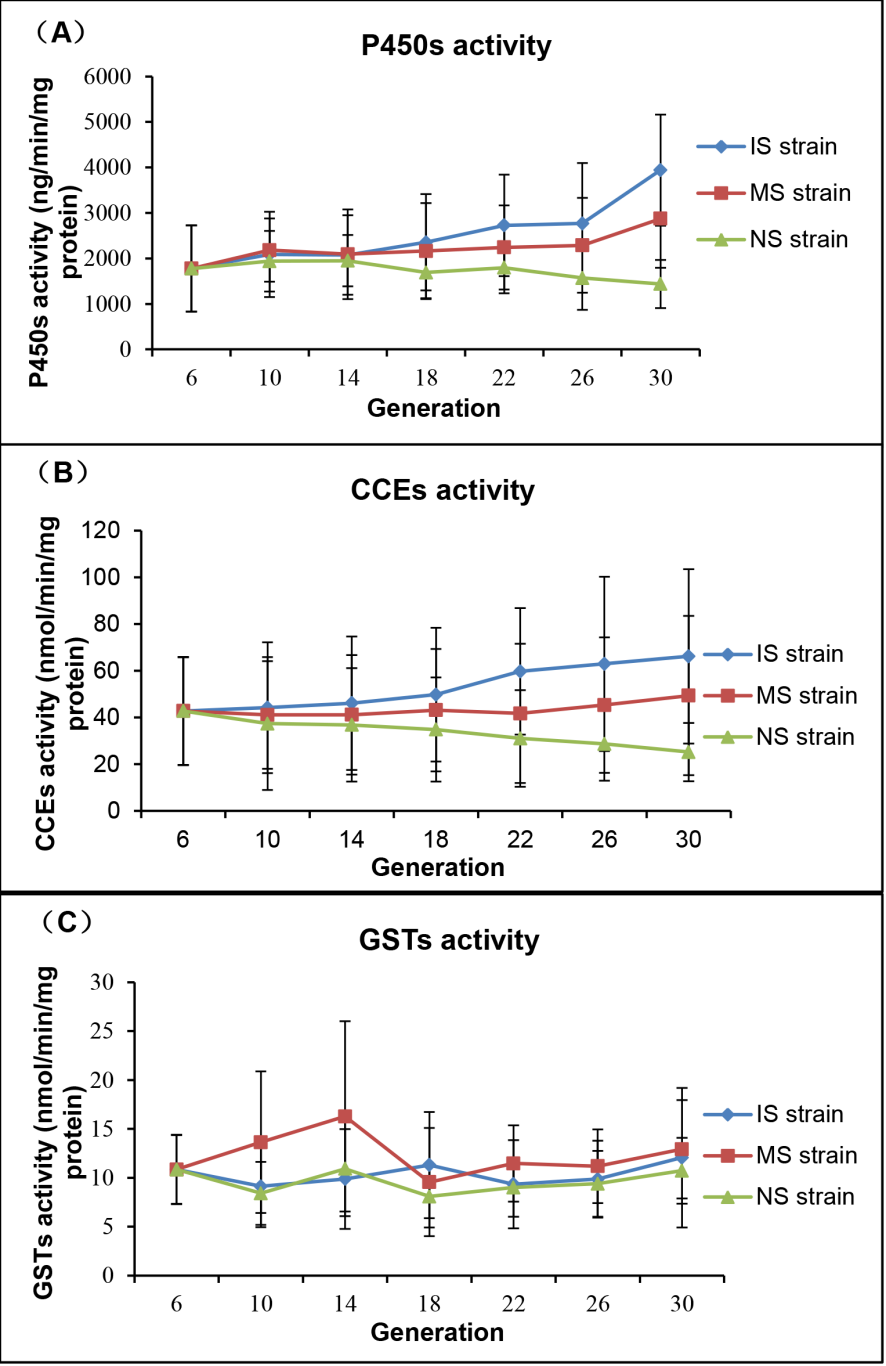
Development of three enzyme activities in populations under different insecticide selection pressures. Dynamic variation of three metabolic enzyme activities under different insecticide selection pressures are shown for (A) P450s, (B) CCEs, and (C) GSTs.

The results showed that the P450s activities of the population changed with different selection conditions (Table 3 and Fig 5). In the IS strain, significant among-population variation in different generations was found (one-way ANOVA, F6, 560 = 31.312, *P* < 0.0001), and the P450s activities increased significantly with the development of deltamethrin resistance. The P450s activities were increased by 2.21-fold at generation 30, and analysis by *t*-test showed significant variation after generation 14 when compared with activities in generation 6. The P450s activities increased more rapidly after generation 26. Likewise in the MS strain, significant among-population variation in different generations was observed (one-way ANOVA, F6, 561 = 8.841, *P* < 0.0001), and P450s activities also increased significantly with the development of deltamethrin resistance, although the increasing trend was slower than that in the IS strain. P450s activities increased by 1.61-fold at generation 30, and analysis by *t*-test showed significant variation in P450s activities after generation 18. Significant among-population variation was seen in different generations of the NS strain as well (one-way ANOVA, F6, 510 = 7.495, *P* < 0.0001). Without deltamethrin selection, P450s activities were reduced at each generation, decreasing by 0.81-fold at generation 30, and a significant reduction in those activities after generation 26 was found by *t*- test analysis.

Changes of CCEs activities were similar to those of P450s (Table 3 and Fig 5). In the IS strain, significant among-population variation in different generations was observed (one-way ANOVA, F6, 491 = 6.957, *P* < 0.0001), and the CCEs activities also increased significantly with the development of deltamethrin resistance. At generation 30, the activities increased by 1.55-fold, and activities of CCEs increased significantly as shown by *t*- test analysis after generation 18 when compared with those in generation 6. In the MS strain, no significant among-population variation in different generations was detected (one-way ANOVA, F6, 503 = 0.710, *P* = 0. 642 > 0.0001). The CCEs activities were not significantly greater at generation 30 (independent samples test, t = 1.178, *P* = 0.241 > 0.05). In the NS strain, significant among-population variation in different generations was found (one-way ANOVA, F6, 450 = 4.151, *P* < 0.0001). Without deltamethrin selection, CCEs activities were also reduced with each generation, decreasing by 0.59-fold at generation 30, and *t*- test analysis showed significant variations after generation 18.

Activities of P450s and CCEs both increased with the level of deltamethrin resistance, and they decreased after withdrawal of deltamethrin. However, changes of GSTs activities were not associated with deltamethrin resistance (Table 3 and Fig 5). The three replicate groups showed similar trends, and the detailed results are shown in S2 Fig. These results indicated that the enhanced activities of P450s and CCEs led to metabolic resistance in the population, and P450s may hold a prominent role in metabolic resistance.

### The correlation of metabolic enzyme activity and the frequency of L1014F with the degree of resistance

**Fig 6:**
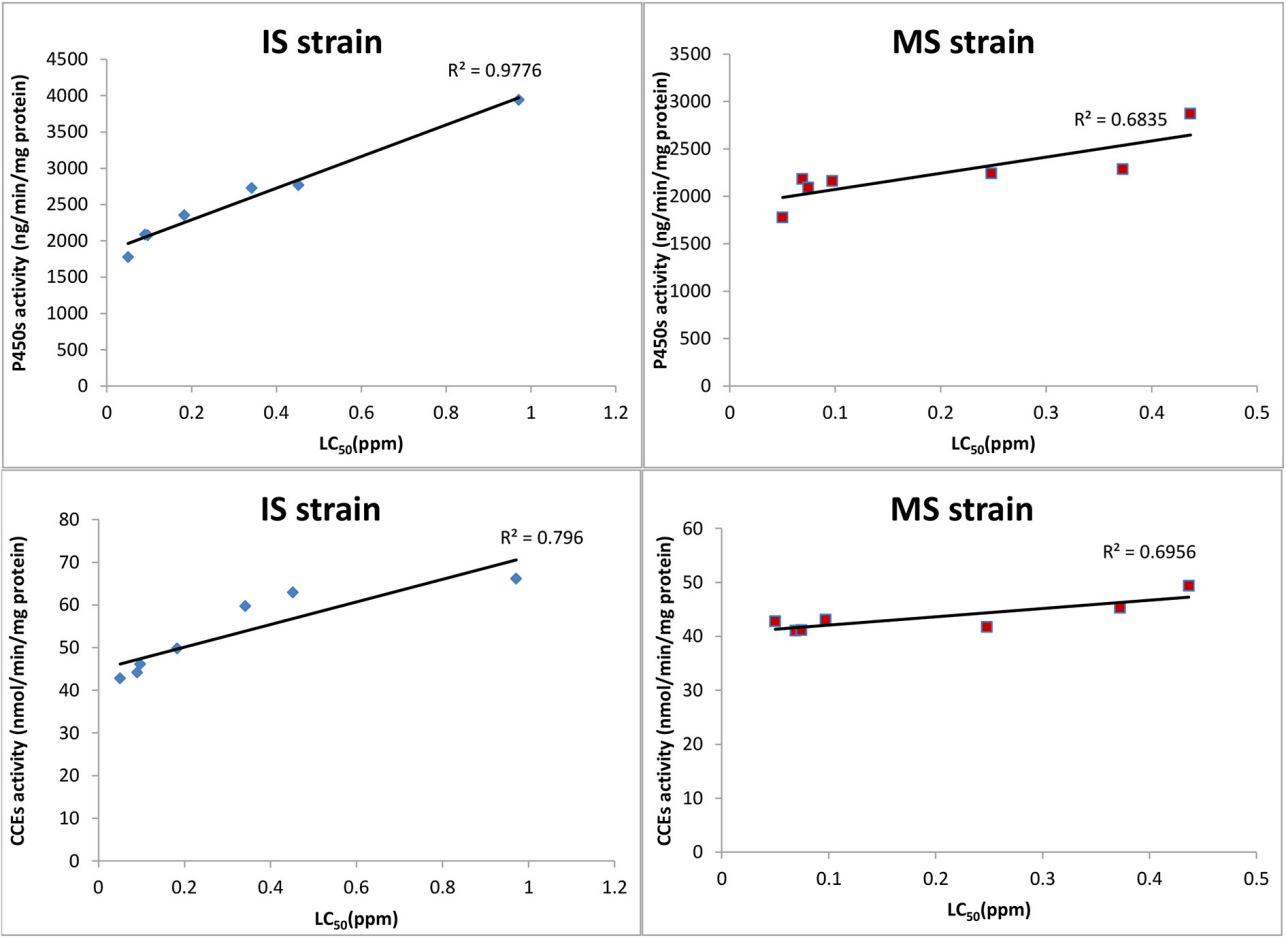
Relationship between metabolic enzyme activity and the degree of resistance in IS strain and MS strain.

Under insecticide selection, a very strong positive correlation was observed between metabolic enzyme activity and the degree of resistance (Table 4 and Fig 6). The activity of P450s and CCEs were significantly correlated. The correlations between metabolic enzyme activity (P450s and CCEs) and LC_50_ in the IS strain were high and slightly higher than correlations in MS strain. We also analyzed the relationship between the frequency of L1014F and the degree of resistance (Table 4). It was noted that the significant correlation between the frequency of L1014F and LC_50_ was only present in MS strain. There were no significant correlations among metabolic enzyme activity, the frequency of L1014F and the degree of resistance in the NS strain.

**Table 4:** Pearson correlation coefficients between metabolic enzyme activity and the frequency of L1014F with the degree of resistance.

	*LC*_*50*_		
	IS	MS	NS
P450s activity	0.989**	0.827*	0.686
CCEs activity	0.892**	0.834*	0.743
GSTs activity	0.543	-0.124	0.075
The frequency of L1014F	0.508	0.811*	0.432

*The correlation coefficients significant *P* < 0.05

** The correlation coefficients significant *P* < 0.01

## Discussion

Resistance of mosquitoes to pyrethroids appears to rely mainly on target-site and metabolic resistance mechanisms. The two mechanisms can occur singly or simultaneously in resistant populations. A growing number of studies have found both metabolic- and *kdr*-based resistance mechanisms in most mosquito species [18, 24, 31]. Most researchers found that metabolic detoxification was the most important mechanism for the development of resistance in the mosquito population, whereas the target site played a less important role [32, 33]. Preliminary investigations of underlying resistance mechanisms of the pyrethroid resistance in field populations of *Anopheles funestus* in southern Africa indicated that a P450-based metabolic resistance was the main mechanism with no *kdr* mutation identified yet in this species [34]. Ochomo *et al*. found that phenotypic resistance to permethrin in *An*. *gambiae s*.*s*. was attributed to elevated expression of β-esterase and oxidase enzymes and the presence of *kdr* alleles at the voltage-gated sodium channel locus, but target-site mechanisms was detected in phenotypic resistance to deltamethrin solely [35]. It was noted that the different mechanisms occurred in the same resistance population. Although studies on the two resistance mechanisms have provided insights, how the target-site and metabolic resistance mechanisms differentially contribute to the resistance phenotype has remained unclear. To tackle these issues, our study detected *kdr* and three detoxification enzyme activities in the same mosquito sample and dynamically monitored the trends of resistance in populations with different insecticide selection pressures. We found that *kdr* and metabolic resistance both contributed to deltamethrin resistance in the *Cx*. *pipiens pallens* populations, but they had different roles under different selection pressures. The P450s activities increased significantly after generation 14 for IS strain and 18 for MS strain, and only the CCEs increased significantly after generation 18 in IS strain. In both the IS and MS groups, resistance to insecticide and frequencies of the *kdr* mutation L1014F increased before the detoxification enzyme activities were significantly increased. This phenomenon indicated that the target-site mechanisms was important under a relatively low insecticide selection pressure. After the population became homozygous for the L1014F mutation, the level of resistance grew along with the increase in detoxification enzyme activities. Linear correlation analysis showed that the significant correlation between the frequency of L1014F and LC_50_ was only present in MS strain, but the correlations between metabolic enzyme activity (P450s and CCEs) and LC_50_ was significantly high both in IS and MS strain, and slightly higher in IS strain. This finding seemed to indicate that the metabolic resistance increased with the increase of selection pressure, and played a main role in causing a high level of resistance under high insecticide selection pressure. Individual organisms with a low fitness cost will survive under selection. Numerous disruptive mutations can confer resistance (whether through suppression, down-regulation or gene-silencing), and a high dose of a selective agent may overcome fitness costs associated with disruption and thus favor a large pool of normally deleterious mutations [36, 37]. However, fitness costs also select against such alleles in the absence of toxicant selection. Metabolic resistance is mainly caused by the up-regulation of detoxification enzymes with high fitness costs. Some studies suggest that P450s overproduction decreases the fitness of individual organisms that carry them because the overproduced P450s can metabolize hormonal endogenous molecules. However, amino-acid substitutions may possibly involve fewer disturbances to the fitness of the individual [38]. Therefore, the low insecticide selection pressure preferred selection of individuals with *kdr*. When the selection pressure was increased via a high dose of insecticide, organisms with metabolic resistance were primarily selected.

Metabolic resistance to pyrethroid is known to be associated with three types of detoxification enzymes, P450s, CCEs and GSTs; however, the relative importance and pattern of these enzymes in the development of resistance are still disputed. Pyrethroid resistance is thought to be mediated essentially by P450s. Elevated levels of P450s activity are frequently observed in mosquito species as major malaria vectors in Africa [11, 39, 40]. In whole-genome microarray studies, members of the cytochrome P450 class of metabolic enzymes are frequently up-regulated in pyrethroid-resistant mosquitoes [41]. CCEs are also believed to act as a cause of metabolic resistance in some instances. Recently, the capacity of *Aedes aegypti* CCEs to metabolize pyrethroids leading to detoxification has been demonstrated *in vitro* [11]. GSTs are regularly found overexpressed in pyrethroid-resistant mosquitos. The potential role of GSTs in pyrethroid resistance is likely associated with protection against oxidative stress and sequestration of pyrethroids [42, 43]. Detoxification of pyrethroids by P450s either alone or in combination with CCEs and/or GSTs has been suggested to play a role in pyrethroid resistance [8, 44, 45]. In the same population, the metabolic resistance was also different. Ochomo *et al*. showed the association of elevated activities of β-esterases and P450s with permethrin resistance, but there was no elevated expression of detoxifying enzymes in phenotypic resistance to deltamethrin [35]. The different conclusions among diverse studies confirmed that the development of metabolic resistance is a rather complicated process, which may be affected by the species, strain (field or lab) or insecticides. In our study, the same species and strain were exposed to the identical insecticide, so we could detect the effects of selection pressure on metabolic resistance. Our study found that different selection pressures led to different levels of metabolic resistance with potentially different mechanisms. Metabolic resistance was mainly mediated by P450s under low insecticide selection pressure, but a high level of metabolic resistance was related to P450s and CCEs under high insecticide selection pressure. Results of the metabolic enzyme assay showed that the development of deltamethrin resistance was accompanied by the significant increase in activities of P450s and CCEs in the IS strain, and only P450s were raised in the MS strain. No correlations between GSTs and the level of deltamethrin resistance were found here. The resistance levels and P450s activity were similar at generation 30 for MS strain and generation 26 for IS strain, but the CCEs activity was significantly elevated in IS strain (Table 3 and S2 Table). CCEs activity appeared higher in IS strain, but CCEs activity made substantial jumps from generation 22 to generation 30 in the MS strain. These results suggested that insecticide selection had a finite amount of genetic variation in the base population that could be selected to generate resistant phenotypes. Under mild selection (in MS strain), the P450s activity responded first, then the CCEs activity was necessary and some of the genetic variants responsible for higher CCEs activity were selected from generations 22 to achieve high levels of resistance. Under intense selection pressure (in IS strain), perhaps P450s alleles alone could not segregate fast enough to produce the required resistance level, so CCEs alleles were selected concurrently. As we did not examine the members of the P450s and CCEs families involved in metabolic resistance, further investigations are needed to identify the associated enzymes and their mechanisms in the metabolic-based resistance.

When genomic regions are subjected to strong and recent selection pressure, the adjacent sequences extending outward from the site of selection with reduced diversity provide evidence of a selective sweep [41, 46]. Our study showed the diversity of the *kdr* gene was reduced with increased resistance under deltamethrin selection by cloning and sequencing the intron region downstream of L1014. These results confirmed that *kdr* led to the deltamethrin resistance in this population. In our study, a higher association of the L1014F mutation than the L1014S mutation was found with deltamethrin resistance in the *Cx*. *pipiens pallens* population. A similar conclusion was made in our previous research [22]. In the current study, we only detected the region around L1014 in the *kdr* gene, and whether other *kdr* mutation alleles were related to pyrethroid resistance in this *Cx*. *pipiens pallens* population was unclear.

Current chemical control programs are largely dependent on pyrethroids, and their efficacy is now threatened by the rise of resistance in target populations. Therefore, formulating a new insecticide use strategy to delay the development and/or spread of pyrethroid resistance is a priority. Based on results of our study, we propose some considerations for insecticide use. It is a must to avoid long-term exposure of mosquito populations to a constant low concentration of insecticide, because it can lead to significantly increased resistance in populations. In this study, when we selected the population (MS strain) with a constant concentration of deltamethrin (0,05ppm) for 24 generations, the level of resistance grew exponentially and the RR was increased more than eight times. This result suggested that the low concentration of insecticide in environment may affect the vector control, such as insecticides used in agriculture. So reducing pesticide residues and accelerating degradation in the environment may delay the development of resistance. Pesticide use should suit to local conditions, because the *k*dr and metabolic resistance had different roles under different selection pressures. Our study found that metabolic resistance mainly played a main role under high insecticide selection pressure. So the combination of insecticide and synergists of metabolic enzymes could delay the increase of insecticide in these areas with high resistance. For example the synergist PBO (piperonyl butoxide), a known inhibitor of P450s and esterase activity, has a significant role in vector control and is encouraged by WHO [47]. The NS strain in our study showed a reduction of RR by ~2-fold after 24 generations, but it was still higher than that at the first generation. This indicated that the high frequency of resistance alleles in a population might result in the slow recovery of sensitivity. The *kdr* alleles were fixed by generation 14 in the NS strain but the RR was still falling. Combined with the results of the steady decline in P450s and CCEs activities (Fig 5), the changes in RR might be caused by reductions in metabolic enzyme activities. However, the RR was still higher than the first generation, it might indicate that fitness costs of metabolic resistance were low. It needs further investigation. Whether in IS or MS, the level of resistance grew exponentially after long term exposure to pesticides. It is a must to rotate the insecticides promptly before the occurrence of high resistance. The insecticides adopted for rotations should have different modes of action to avoid cross-resistance. We demonstrated that the *kdr* gene and metabolic enzymes played different roles in the development of pyrethroid resistance. Hence, the target sites of insecticides and the resistance induced by metabolic enzymes should both be taken into account in rotations. In addition, as the pattern of decline in resistance can be slow, insecticide withdrawal must be maintained for an extended period of time. A study by Raghavendra *et al*. showed that persistence of resistance to DDT and malathion was respectively observed 30 yr and 9 yr after withdrawal from IRS, although reversal of deltamethrin resistance was observed relatively rapidly within 2–3 yr after its withdrawal from IRS [48]. Conclusions from our study may provide a reference for future management of insecticide resistance in mosquitoes and other arthropod pests and disease vectors.

## Supporting Information

S1 Fig Dynamics changes of *kdr* allele frequencies in the strains at each generation under different insecticide selection pressures. Dynamic changes of *kdr* allele frequencies are shown for (A) IS strain, (B) MS strain, and (C) NS strain. Ⅰ, Ⅱ and Ⅲ represent the three replicate groups.
(PDF)

S2 Fig Development of activities of three enzymes in three replicate groups at each generation under different insecticide selection pressures. (A) Dynamic changes of P450 activities at each generation in IS, MS and NS strains. (B) Dynamic changes of CCE activities at each generation in IS, MS and NS strains. (C) Dynamic changes of GSTs activities at each generation in IS, MS and NS strains. Ⅰ, Ⅱ and Ⅲ represent the three replicate groups.
(PDF)

S1 Table The exposure dose used for deltamethrin selection per generation. (DOC)

S2 Table LC_50_ values in populations under different insecticide selection pressures. *Resistance ratio is the ratio of LC_50_ of the test generation to LC_50_ of the S-LAB. S-LAB: laboratory deltamethrin-susceptible strain of *Cx*. *pipiens pallens*.
(DOC)
