# Supplementary figures and images for: Development of Resistance to Pyrethroid in Culex pipiens pallens Population under Different Insecticide Selection Pressures
Source: PLoS Negl Trop Dis. 2015 Aug 14;9(8):e0003928. doi: 10.1371/journal.pntd.0003928 (PMC4537097; doi:10.1371/journal.pntd.0003928)

(A)

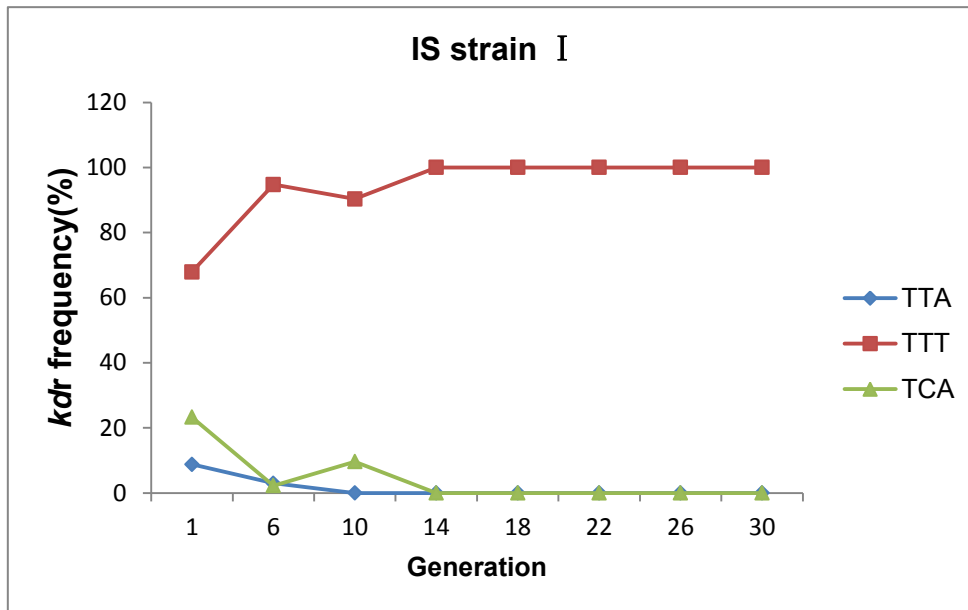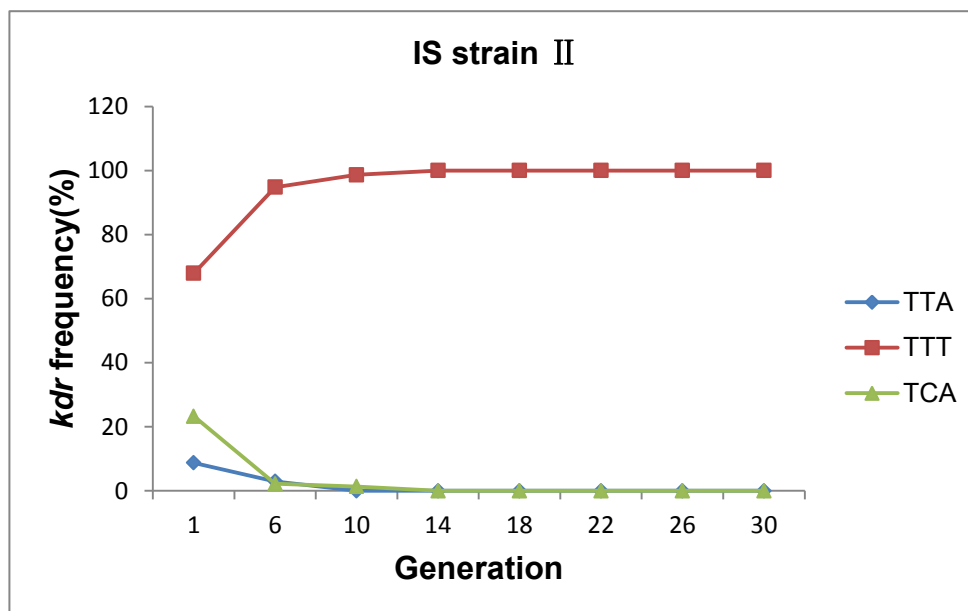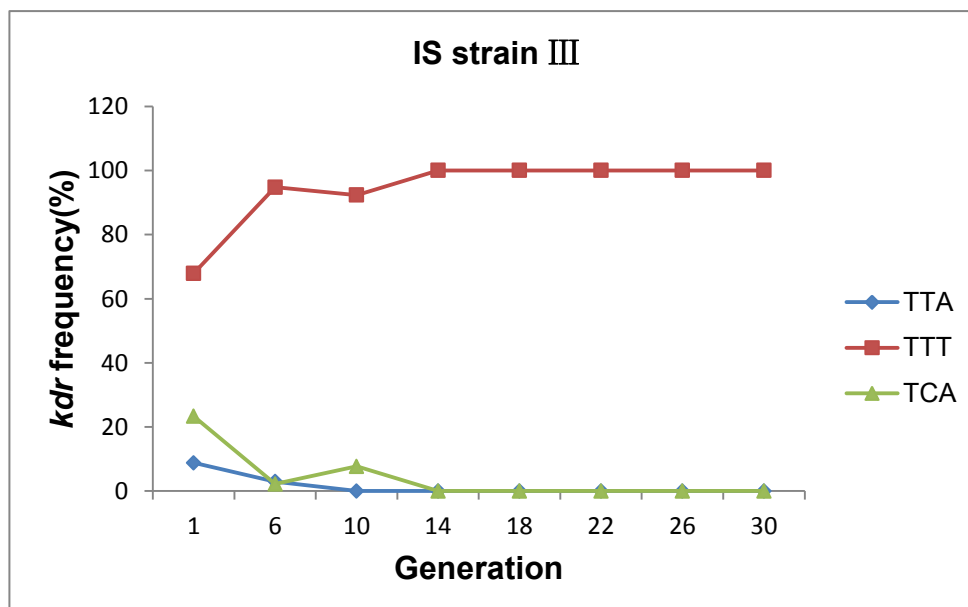

(B)

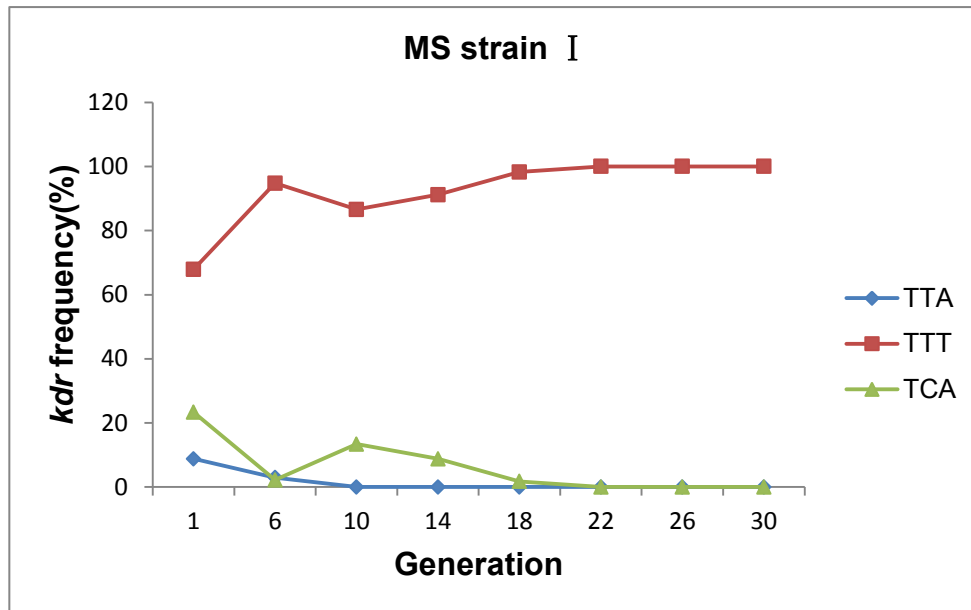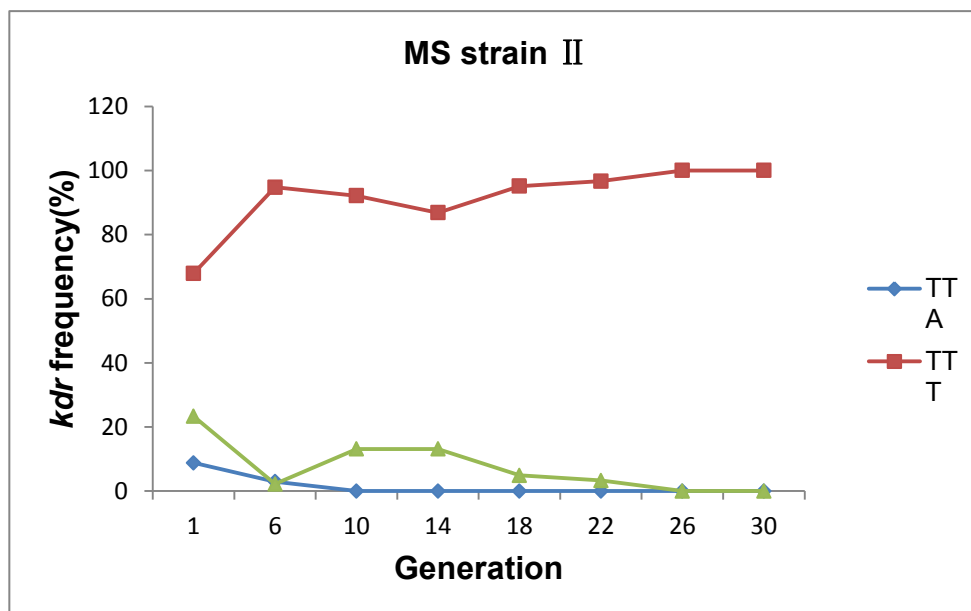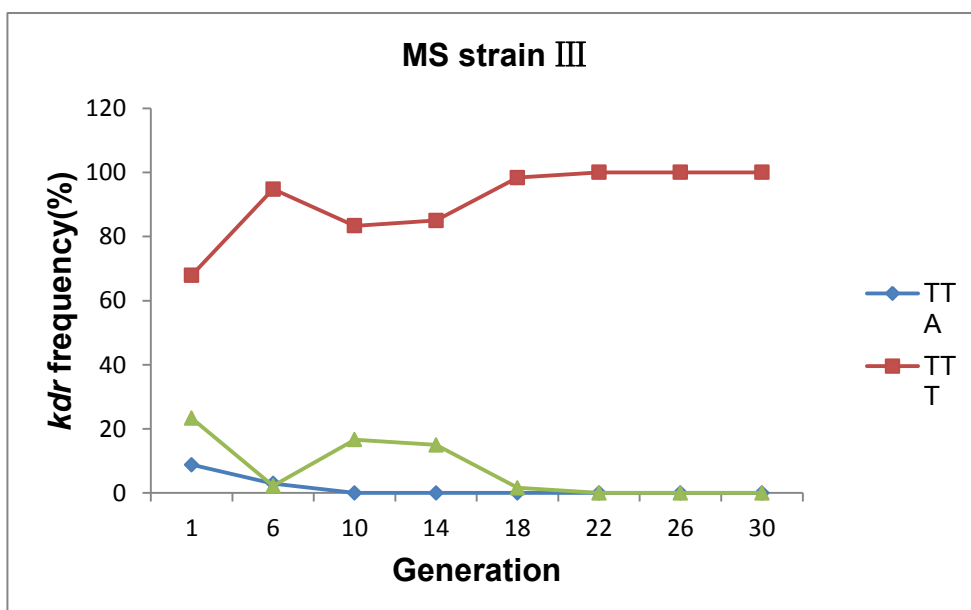

(C)

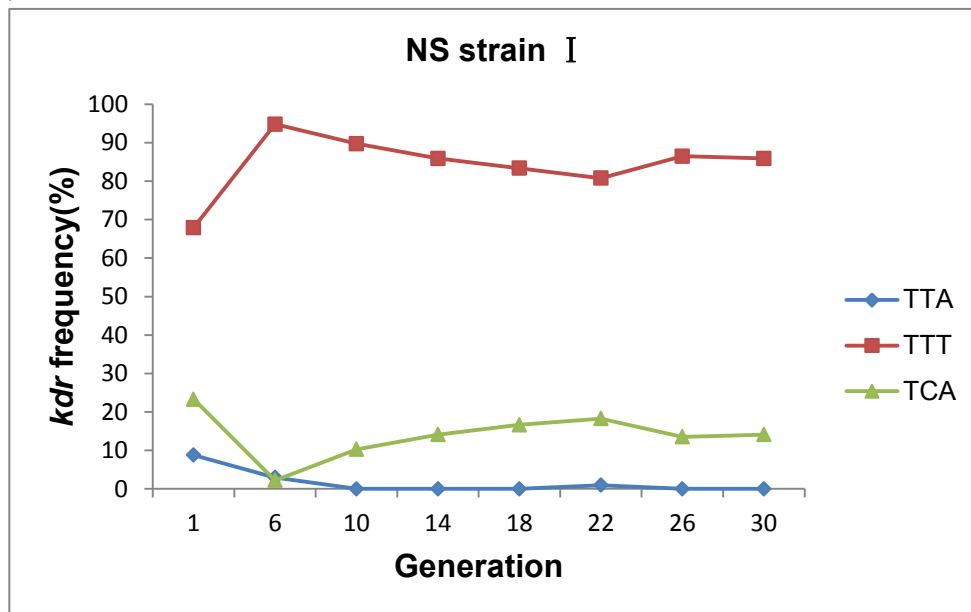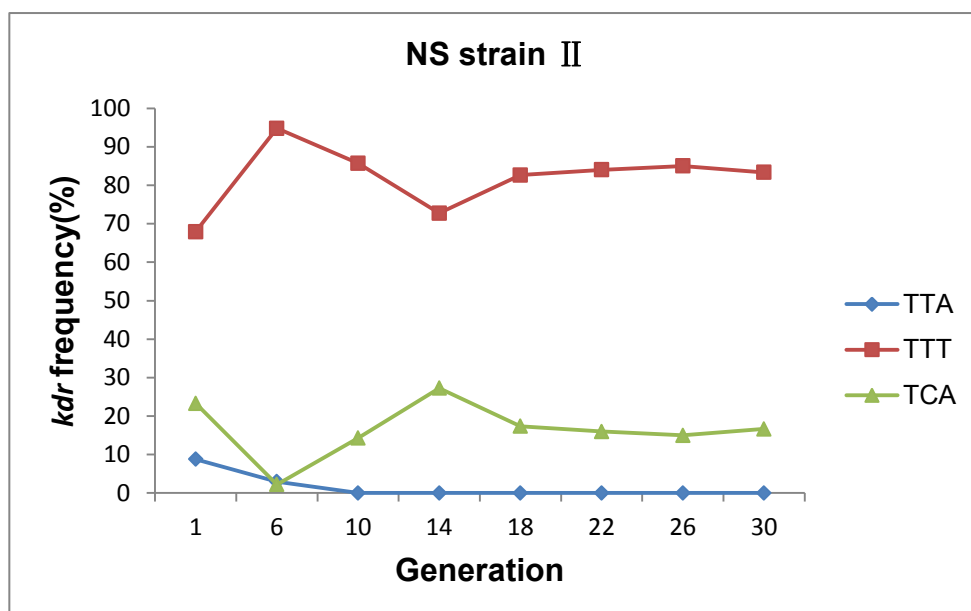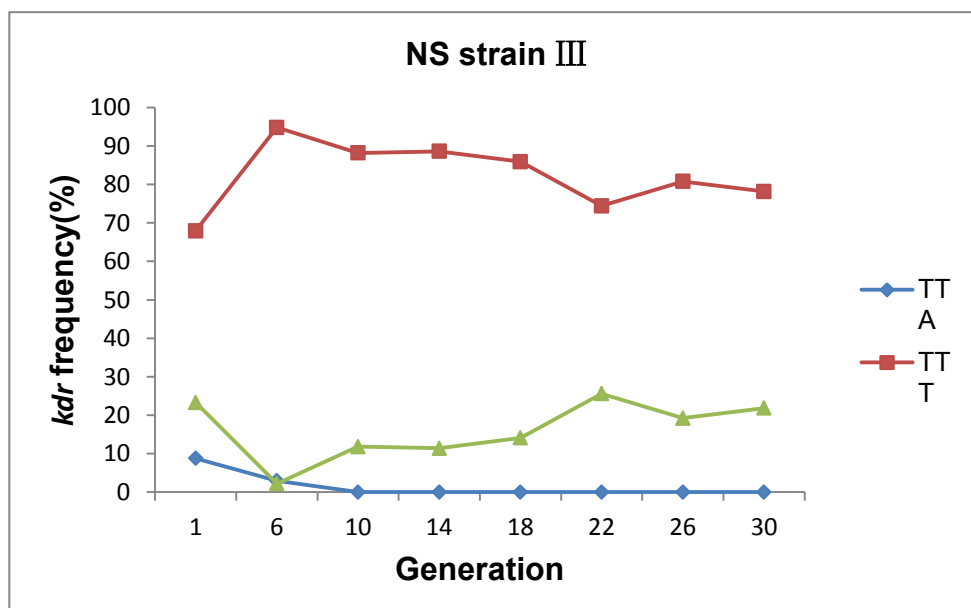

Supplement: S1 Fig — Dynamic changes of kdr allele frequencies are shown for (A) IS strain, (B) MS strain, and (C) NS strain. Ⅰ, Ⅱ and Ⅲ represent the three replicate groups. (PDF) [file pntd.0003928.s001.pdf]

(A)

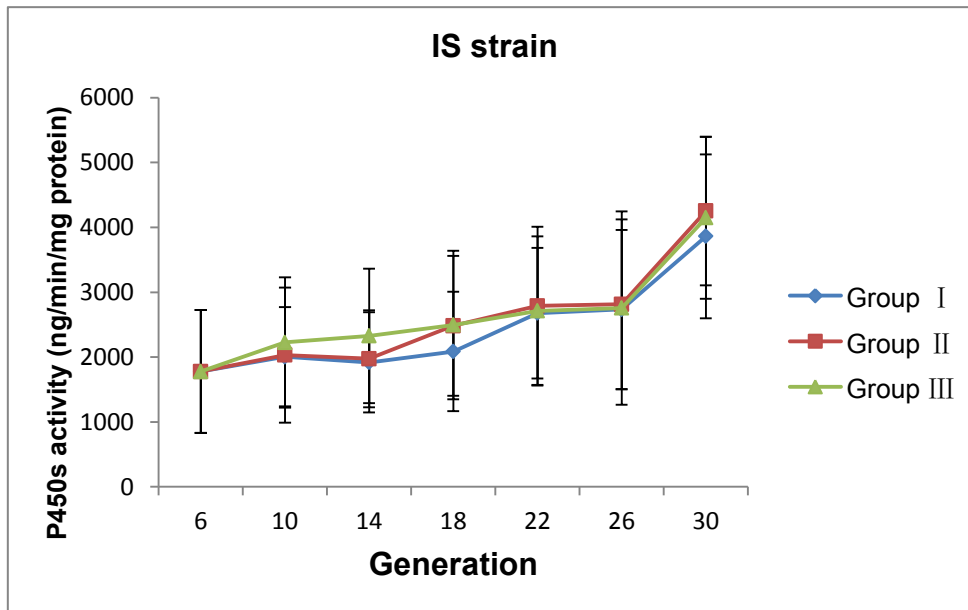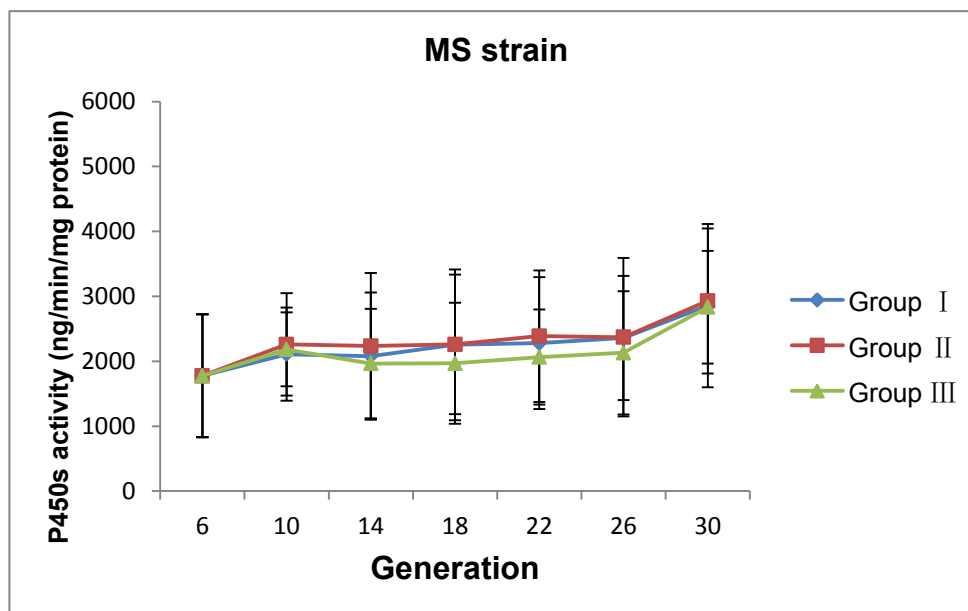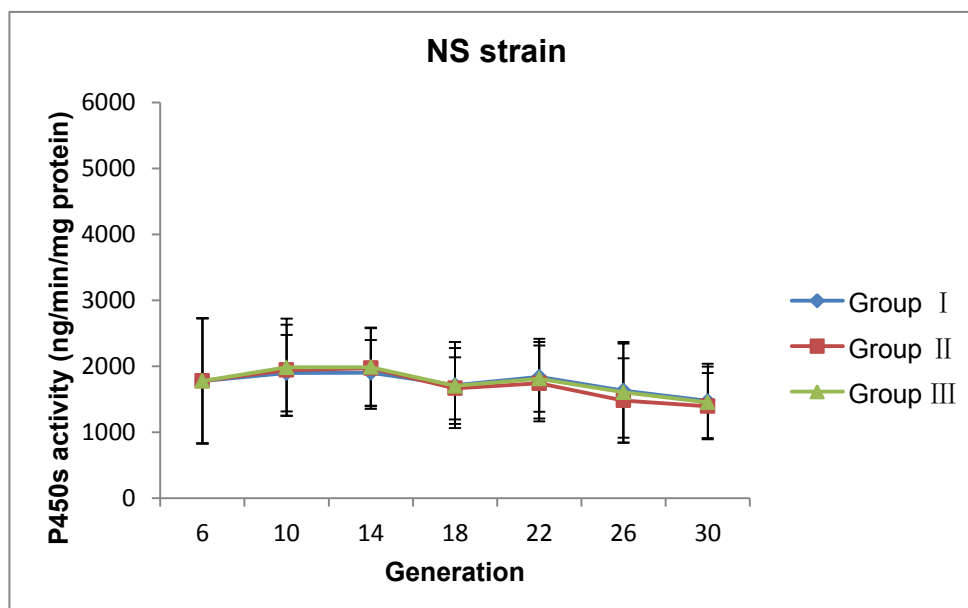

(B)

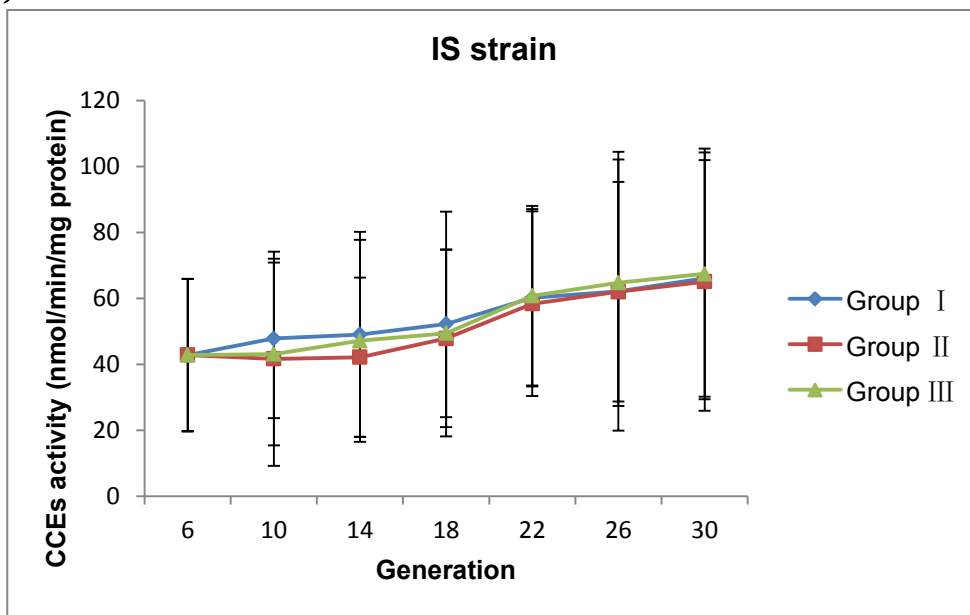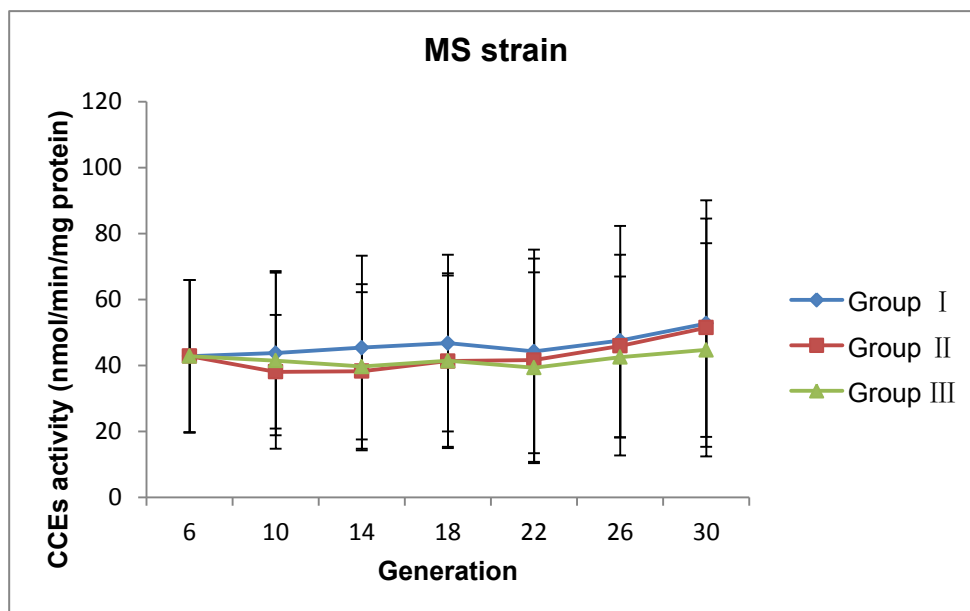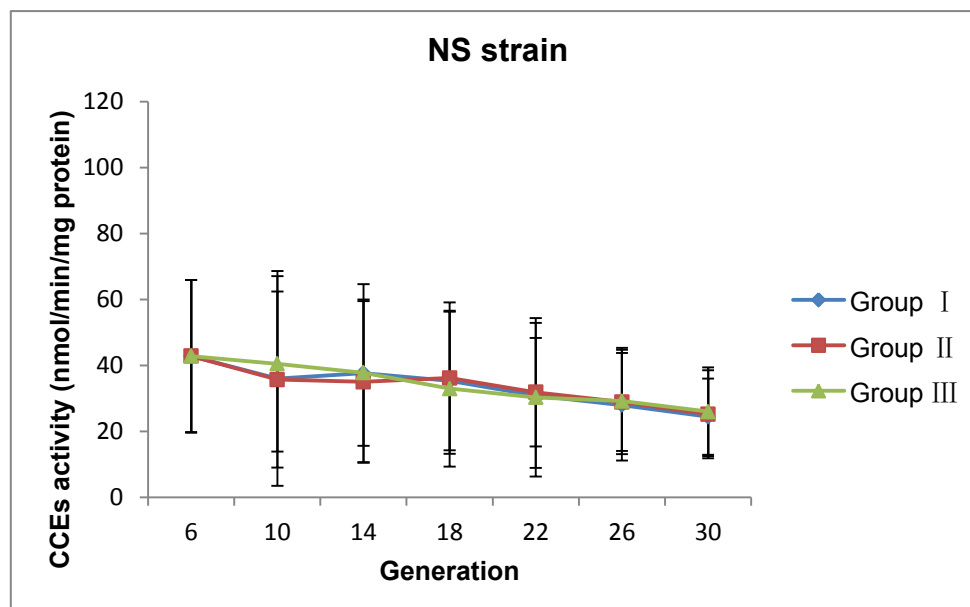

(C)

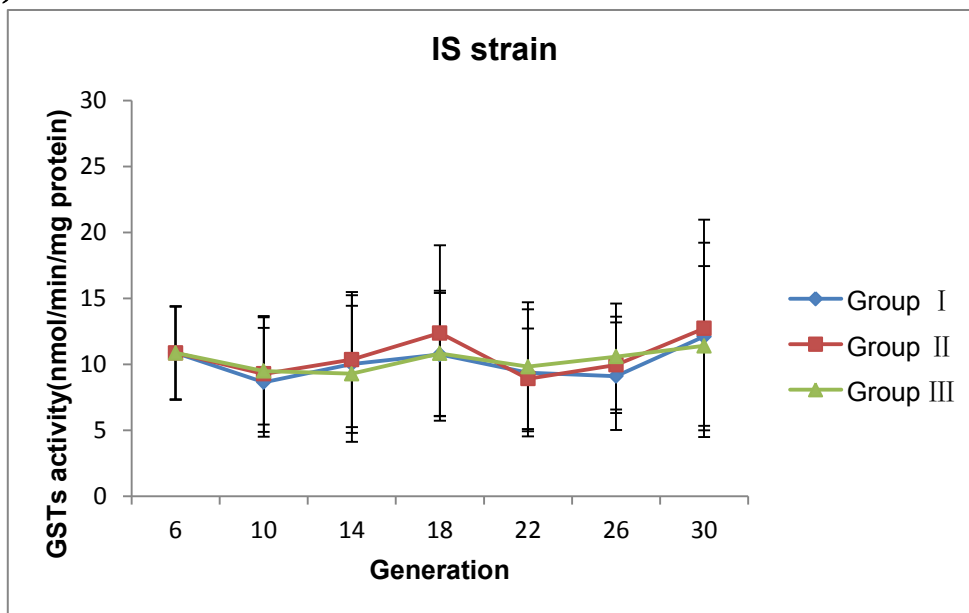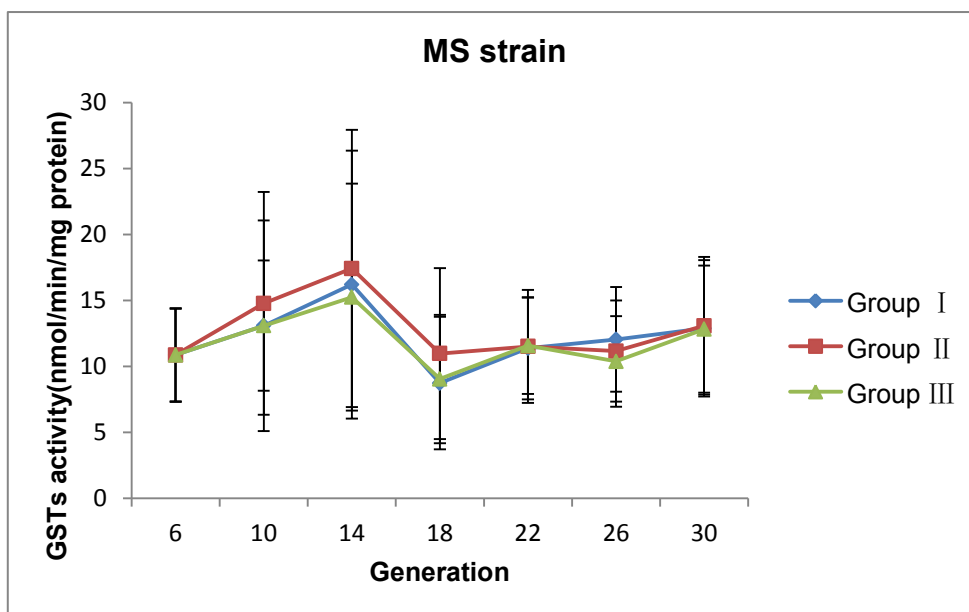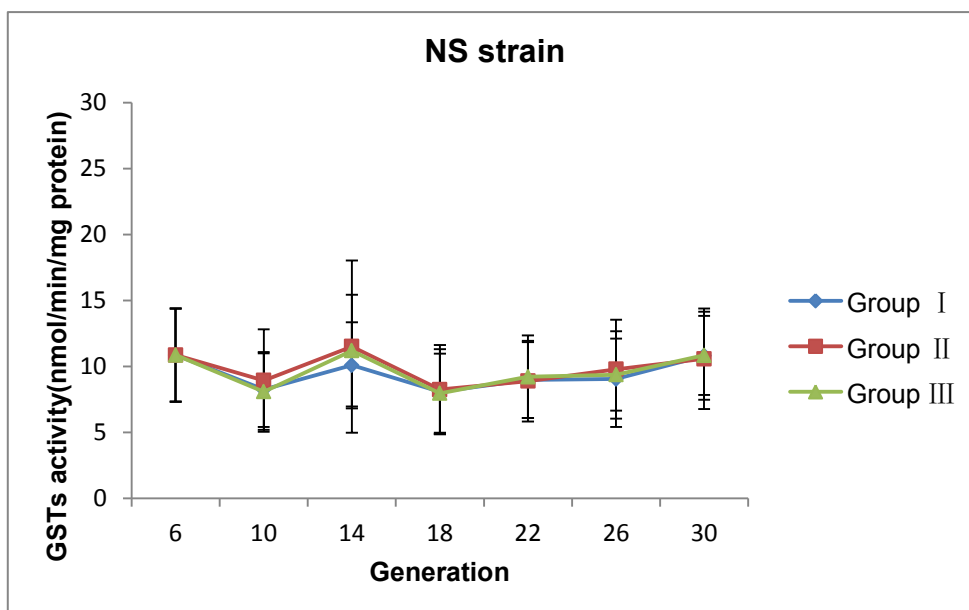

Supplement: S2 Fig — (A) Dynamic changes of P450 activities at each generation in IS, MS and NS strains. (B) Dynamic changes of CCE activities at each generation in IS, MS and NS strains. (C) Dynamic changes of GSTs activities at each generation in IS, MS and NS strains. Ⅰ, Ⅱ and Ⅲ represent the three replicate groups. (PDF) [file pntd.0003928.s002.pdf]
