# Supplementary material for: Development of Resistance to Pyrethroid in Culex pipiens pallens Population under Different Insecticide Selection Pressures
Source: PLoS Negl Trop Dis. 2015 Aug 14;9(8):e0003928. doi: 10.1371/journal.pntd.0003928 (PMC4537097; doi:10.1371/journal.pntd.0003928)
Supplement: S1 Table — (DOC) [file pntd.0003928.s003.doc]

| ***Strains*** | ***Generation*** | ***The exposure dose（ppm）*** |
| --- | --- | --- |
| **Lab selection** | 1 | 0.02 |
|  | 2 | 0.03 |
|  | 3 | 0.03 |
|  | 4 | 0.04 |
|  | 5 | 0.05 |
|  | 6 | 0.05 |
| **IS strain** | 7 | 0.06 |
|  | 8 | 0.10 |
|  | 9 | 0.09 |
|  | 10 | 0.09 |
|  | 11 | 0.09 |
|  | 12 | 0.09 |
|  | 13 | 0.09 |
|  | 14 | 0.10 |
|  | 15 | 0.10 |
|  | 16 | 0.10 |
|  | 17 | 0.15 |
|  | 18 | 0.18 |
|  | 19 | 0.20 |
|  | 20 | 0.25 |
|  | 21 | 0.30 |
|  | 22 | 0.34 |
|  | 23 | 0.40 |
|  | 24 | 0.42 |
|  | 25 | 0.44 |
|  | 26 | 0.45 |
|  | 27 | 0.58 |
|  | 28 | 0.76 |
|  | 29 | 0.85 |
|  | 30 | 0.97 |
| **MS strain** | 7−30 | 0.05 |
