# Supplementary material for: Development of Resistance to Pyrethroid in Culex pipiens pallens Population under Different Insecticide Selection Pressures
Source: PLoS Negl Trop Dis. 2015 Aug 14;9(8):e0003928. doi: 10.1371/journal.pntd.0003928 (PMC4537097; doi:10.1371/journal.pntd.0003928)
Supplement: S2 Table — *Resistance ratio is the ratio of LC50 of the test generation to LC50 of the S-LAB. S-LAB: laboratory deltamethrin-susceptible strain of Cx. pipiens pallens. (DOC) [file pntd.0003928.s004.doc]

| ***Strains*** | ***Generation*** | ***LC_50_ (ppm)*** | ***95% CI*** | ***Slope（±SE）*** | ***Resistance ratio**** |
| --- | --- | --- | --- | --- | --- |
| **S-LAB** |  | 0.0122 | 0.0065-0.0118 | 1.7349±0.0029 | 1 |
| **Lab selection** | 1 | 0.0206 | 0.0151-0.0281 | 2.1335±0.0033 | 1.69 |
|  | 6 | 0.0501 | 0.0354-0.0710 | 1.7960±0.0089 | 4.11 |
| **IS strain** | 10 | 0.09 | 0.0584-0.1385 | 1.7563±0.0198 | 7.38 |
|  | 14 | 0.0968 | 0.0711-0.1317 | 2.1012±0.0152 | 7.93 |
|  | 18 | 0.1827 | 0.1161-0.2877 | 1.6653±0.0423 | 14.98 |
|  | 22 | 0.3414 | 0.2162-0.5392 | 1.5791±0.0796 | 27.98 |
|  | 26 | 0.452 | 0.3268-0.6252 | 1.9938±0.0748 | 37.05 |
|  | 30 | 0.9713 | 0.7536-1.2699 | 2.6338±0.1302 | 79.61 |
| **MS strain** | 10 | 0.069 | 0.0508-0.0936 | 2.4166±0.0107 | 5.66 |
|  | 14 | 0.0745 | 0.0534-0.1040 | 2.2099±0.0126 | 6.11 |
|  | 18 | 0.0973 | 0.0719-0.1318 | 2.1510±0.0151 | 7.98 |
|  | 22 | 0.2479 | 0.1431-0.4293 | 1.7110±0.0694 | 20.32 |
|  | 26 | 0.3725 | 0.2466-0.5627 | 1.9545±0.0784 | 30.53 |
|  | 30 | 0.4367 | 0.2677-0.7124 | 1.7438±0.1090 | 35.80 |
| **NS strain** | 10 | 0.0531 | 0.0390-0.0723 | 2.1012±0.0084 | 4.35 |
|  | 14 | 0.0511 | 0.0376-0.0696 | 2.1012±0.0081 | 4.19 |
|  | 18 | 0.0517 | 0.0380-0.0703 | 2.1012±0.0082 | 4.24 |
|  | 22 | 0.0426 | 0.0303-0.0598 | 1.8921±0.0074 | 3.49 |
|  | 26 | 0.037 | 0.0258-0.0531 | 1.8614±0.0068 | 3.03 |
|  | 30 | 0.0241 | 0.0180-0.0322 | 2.2525±0.0036 | 1.98 |

*Resistance ratio is the ratio of LC_50_ of the test generation to LC_50_ of the S-LAB. S-LAB: laboratory deltamethrin-susceptible strain of *C. pipiens pallens*.
